# *Aedes albopictus* and *Aedes flavopictus* (Diptera: Culicidae) pre-imaginal abundance patterns are associated with different environmental factors along an altitudinal gradient

**DOI:** 10.1016/j.cris.2020.100001

**Published:** 2020-10-15

**Authors:** Luis Fernando Chaves, Mariel D. Friberg

**Affiliations:** a Instituto Costarricense de Investigación y Enseñanza en Nutrición y Salud (INCIENSA), Apartado Postal 4-2250, Tres Ríos, Cartago, Costa Rica; b Earth Science Division, NASA Goddard Space Flight Center, Greenbelt, MD 20771, USA; c Universities Space Research Association, Columbia, MD 21046, USA

**Keywords:** Schmalhausen`s law, Invasive species, Environmental gradients, NDVI and EVI, Zero inflated count models

## Abstract

*Aedes (Stegomyia) albopictus* (Skuse) is a major global invasive mosquito species that, in Japan, co-occurs with *Aedes (Stegomyia) flavopictus* Yamada, a closely related species recently intercepted in Europe. Here, we present results of a detailed 25-month long study where we biweekly sampled pupae and fourth instar larvae of these two species from ovitraps set along Mt. Konpira, Nagasaki, Japan. This setting allowed us to ask whether these species had different responses to changes in environmental variables along the altitudinal gradient of an urban hill. We found that spatially *Ae. albopictus* abundance decreased, while *Ae. flavopictus* abundance increased, the further away from urban land. *Ae. flavopictus* also was more abundant than *Ae. albopictus* in locations with homogenous vegetation growth with a high mean Enhanced Vegetation Index (EVI), platykurtic EVI, and low SD in canopy cover, while *Ae. albopictus* was more abundant than *Ae. flavopictus* in areas with more variable (high SD) canopy cover. Moreover, *Ae. flavopictus* abundance negatively impacted the spatial abundance of *Ae. albopictus*. Temporally we found that *Ae. flavopictus* was more likely to be present in Mt. Konpira at lower temperatures than *Ae. albopictus*. Our results suggest that spatial and temporal abundance patterns of these two mosquito species are partially driven by their different response to environmental factors.

## Highlights

• *Aedes flavopictus* is a mosquito species that has been recently intercepted in Europe.
• In Japan *Ae flavopictus* co-occurs with *Aedes albopictus,* a globally invasive mosquito species.
• *Ae. flavopictus* was a rare species, but now is common in places where it was absent and *Ae. albopictus* was present.
• Spatially we found *Ae. albopictus* in locations close to urban land use while *Ae. flavopictus* preferred land with vegetation.
• *Ae. flavopictus* aquatic abundance could increase in colder and more platykurtic environments.

## Introduction

Similar species often compete for the same resources, yet they are able to co-exist. Macarthur and Levins (1967) proposed that species co-existence can emerge under conditions where there is a limiting similarity over which species that exploit a common pool of resources diverge in resource use, for example, by having a different response to uncertainties, and/or gradients, in the environment. Indeed, organisms cope with different patterns of environmental variability (Levins, 1968). For example, there is temporal variability that can have periodic and predictable characteristics, as observed in seasonal environments (Stearns, 1981), there is spatial variability generated by natural environmental gradients, like those arising from altitudinal differences (Hodkinson, 2005), while some of the variability, both temporal and spatial, might be better described by the contingency associated with the distribution of random variables (Chaves et al., 2012, 2014). Therefore, organisms, beyond following an average environment, also respond to higher-order moments, *e.g.,* SD and kurtosis, of change in the environment, a biological principle that has been called Schmalhausen's law (Chaves, 2017a; Chaves and Koenraadt, 2010). Thus, based on species limiting similarity and Schmalhausen's law, our hypothesis is that abundance patterns of related co-existing species are associated with different responses to the same set of environmental variables, where environmental variables are measured considering their mean values, but also higher order moments in their statistical distribution along a natural gradient. Here, we test this hypothesis by looking at the spatial and temporal abundance patterns of the globally invasive Asian tiger mosquito, *Aedes (Stegomyia) albopictus* (Skuse), and a closely related species, *Aedes (Stegomyia) flavopictus* Yamada, along an altitudinal gradient in their native range.

Early mosquito studies in Japan reported that *Ae. albopictus* commonly co-occurred with *Ae. flavopictus*, a morphologically similar species (Yamada, 1921). Several mosquito surveys have shown that both species are present in the Korean Peninsula and throughout vast areas of Japan (Kamimura, 1968; La Casse, 1948; Lee, 1987; Maekawa et al., 2016b; Omori et al., 1952; Omori and Sakakibara, 1951; Tanaka et al., 1979). Despite striking morphological similarities, *Ae. albopictus* and *Ae. flavopictus* can be primarily distinguished in the adult stage by the presence of a “patch of narrow curved or crescent-shaped slightly yellowish to yellowish-brown scales” near the wing root (Tanaka et al., 1979). In addition, these two species can be separated based on mitochondrial DNA restriction fragment length polymorphisms (Kambhampati and Rai, 1991), isoenzymes (Gaunt et al., 2004), ITS-1 and ITS-2 ribosomal DNA (Toma et al., 2002, Tsuda et al., 2015) and mitochondrial COI DNA barcoding (Maekawa et al., 2016a; Taira et al., 2012). Therefore, molecular and morphological differences, have robustly confirmed that *Ae. flavopictus* is a different species from *Ae. albopictus,* yet they are phylogenetically the most closely related species to each other in Japan (Sota and Mogi, 2006; Taira et al., 2012). These two species are also unlikely to intermix in nature but able to procreate limited fitness progeny when forced to mate in the laboratory (Miyagi and Toma, 1989).

*Ae. albopictus* is a major invasive vector species that has spread worldwide over recent years (Benedict et al., 2007; Hawley, 1988; Reiter and Darsie, 1984). *Ae. albopictus* likely originated in S.E. Asia and spread from there as a product of increased global commodity trade (Benedict et al., 2007; Reiter and Darsie, 1984). *Ae. albopictus* has been widely recorded over subtropical areas of Japan, as documented by one of the first comprehensive natural history studies on this species (Yamada, 1921). However, historical occurrence records suggest *Ae. albopictus* is spreading into more northern temperate areas of Japan that have seen a temperature rise associated with global warming (Kamimura, 1968; Kobayashi et al., 2002; Maekawa et al., 2016b; Mogi and Tuno, 2014). The expansion of this species toward more northern latitudes is a phenomenon also observed in the New World (Giordano et al., 2020) and Europe (Kuhlisch et al., 2018), leading to predictions about an increased global distribution with warmer temperatures (Campbell et al., 2015; Proestos et al., 2015). Meanwhile, *Ae. flavopictus* was recently intercepted in the Netherlands and it was suggested that it might become an invasive species in Europe (Ibáñez-Justicia et al., 2019), if not already an invasive species there and elsewhere that *Ae. albopictus* has been detected, given its marked morphological similarity with *Ae. albopictus* (Chaves, 2016).

*Ae. albopictus* and *Ae. flavopictus* belong to the subgenus *Stegomyia*, a taxon that also includes several mosquito species known to be competent for the transmission of many major arboviruses affecting humans (Marcondes and Ximenes, 2016), most notably *Aedes (Stegomyia) aegypti* L., the most important dengue vector at a global scale (Whitehorn et al., 2015) and the most common vector of several emerging arboviruses, like chikungunya, Zika and Mayaro (Weaver and Reisen, 2010). *Ae. albopictus* (Gubler and Rosen, 1976; Whitehorn et al., 2015) and *Ae. flavopictus* (Eshita et al., 1982; Srisawat et al., 2016) are both vectorially competent to transmit dengue virus, with evidence suggesting that *Ae. albopictus* is becoming an important dengue vector across the globe (Lambrechts et al., 2010). This highlights the medical importance of both *Ae. albopictus* and *Ae. flavopictus,* granting both species public health importance across their native range, especially in light of both historic (Hotta, 1953) and recent (Kutsuna et al., 2015; Tsuda et al., 2016) dengue epidemics in Japan and the frequent importation of dengue cases into Japan and South Korea (Jeong et al., 2016).

Historic mosquito surveys suggested that in western Japan, *Ae. albopictus* was more widespread than *Ae. flavopictus* (Kamimura, 1968; La Casse, 1948). *Ae. flavopictus* was extremely rare in Nagasaki prefecture in 1948–1949. The few adults found in this prefecture were in a wood near the hot spring resort town of Obama (Omori et al., 1952), 30 km east from Nagasaki City. This Nagasaki prefecture mosquito survey also showed that *Ae. albopictus* was a common, widespread species considering samples were collected all over Nagasaki prefecture (Omori et al., 1952). Nevertheless, a recent study showed that *Ae. flavopictus* has become the most common adult mosquito caught by net sweeping (Chaves, 2016) at Nagasaki city locations where the species was previously not reported (Tsuda et al., 1994; Zea Iriarte et al., 1991). A similar phenomenon, where *Ae. flavopictus* has become common in areas where it used to be rare, has been reported in Shikoku island (Shiraishi, 2011; Yamauchi, 2010), at the same latitude of Nagasaki prefecture in Western Japan, and Toyama prefecture (Yamauchi, 2013), at a slightly more northern latitude than Nagasaki prefecture in Central Japan. Classical studies on the larval ecology of *Ae. flavopictus* showed this species was the most abundant in aged *Phyllostachys edulis* (Carrière) bamboo stumps (Kurihara, 1958) from Sendai city in Miyagi prefecture*.* Additional observations of *Ae. flavopictus* outnumbering *Ae. albopictus* in *Phyllostachys reticulata* (Rupr.) bamboo stumps at Utsunomiya city, in Tochigi prefecture (Kurashige, 1961a, Kurashige, 1961b, b) and the suggestion that these species might be either having antagonistic interactions or different responses to changing environments (Chaves, 2016), and different synchrony patterns (Chaves, 2017b; Chaves et al., 2020), highlight the need to understand which environmental conditions modulate the abundance patterns of these two mosquito species.

Previous mosquito studies at Mt. Konpira, Nagasaki City, Japan, have shown that ovitraps successfully mimic small tree holes, as both have an identical mosquito species composition (Tsuda et al., 1994; Zea Iriarte et al., 1991). Thus, rendering Mt. Konpira ideal for field studies using ovitraps as a proxy of small natural tree holes (*i.e.,* those with a volume below 500 ml) to study pre-imaginal abundance patterns of mosquito species. In addition, the abundance of temporally frequent high (30 m for Landsat 8) and medium (250 m for Moderate Resolution Image Spectroradiometer, MODIS) resolution satellite images at Mt. Konpira (Chaves et al., 2019), allow to estimate time series of vegetation growth indices, such as the Normalized Difference Vegetation Index (NDVI) and the Enhanced Vegetation Index (EVI), which have been related with spatial patterns of insect vector abundance (Kitron et al., 1996) and mosquito population dynamics (Hurtado et al., 2018; Poh et al., 2019; Rigg et al., 2019). Thus, Mt. Konpira also provides a unique opportunity for evaluating the use of satellite derived data, of different spatial resolution, to explain differences in the distribution and abundance of related mosquito species. Moreover, the altitudinal gradient of Mt. Konpira, one of the highest mountains in Nagasaki City (Hoshi et al., 2017), generates environmental and landscape gradients that allows the identification of environmental and landscape conditions favoring the presence and abundance of mosquito species (Chaves et al., 2019, 2015; Chaves and Moji, 2018). Here, we present results from a two-yearlong study where we recorded *Ae. albopictus* and *Ae. flavopictus* pre-imaginal abundance patterns in ovitraps located along the altitudinal gradient of Mt. Konpira, Nagasaki city, Japan. We asked if differences in the association of both species with land use, landscape characteristics, weather variables and remotely sensed changes in vegetation growth might explain their spatial and temporal abundance patterns according to predictions from the limiting similarity of species hypothesis and Schmalhausen's Law.

## Materials and methods

### Study site

**Fig. 1:**
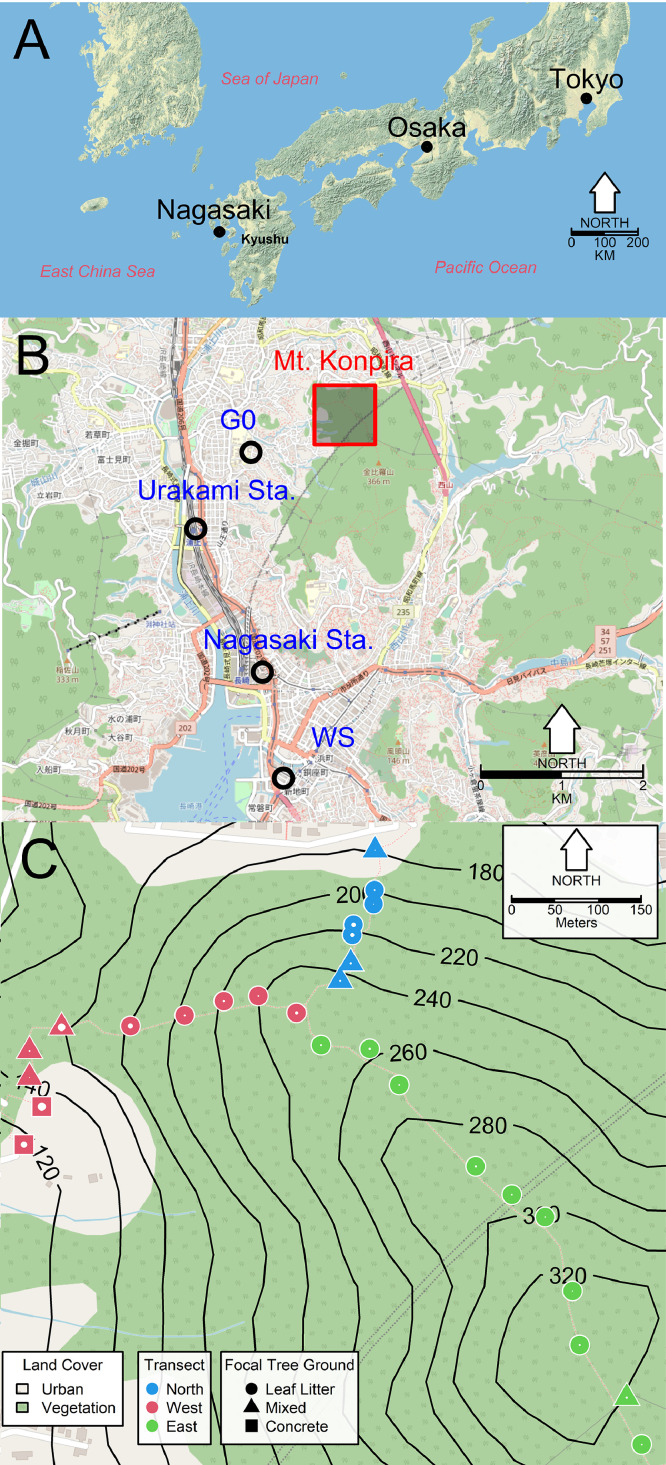
Study Site (A) Nagasaki location within Kyushu island, west of Osaka and Tokyo the two main cities in Japan (B) Nagasaki city highlighting the location of Mt. Konpira in relation to Nagasaki and Urakami train stations, Nagasaki city weather station, WS, and World War II atomic bomb point of impact, G0. The highlighted area enclosed the location of the ovitraps (C) Ovitrap locations at Mt. Konpira, isoclines show the height in m. Isoclines are based on an ASTER digital elevation model, with a 10 m resolution developed by JAXA (available at http://gdem.ersdac.jspacesystems.or.jp/). The inset legend identify urban vs*.* vegetation land use, ground type (character type), and color for the sampling transect. White dots inside each character proportionally represent canopy openness. The base image for map A was courtesy of the US National Park Service (Available at https://www.nps.gov/subjects/hfc/index.htm). The base image for maps B and C were courtesy of © OpenStreetMap (Available at www.openstreetmap.org) under the Open Database License (http://opendatacommons.org/licenses/odbl/1.0/).

The study site was Mt. Konpira, a hill in Nagasaki, a medium-sized city, with less than 500,000 people and around 1000 inhabitants/km^2^ (Chaves and Moji, 2018), in western Japan (Fig. 1A). Mt. Konpira is embedded within the urban landscape of Nagasaki city, where it is surrounded by human built environment, primarily used for housing, and close to major historical landmarks, most notably the impact site of the Nagasaki atomic bomb during World War II (Fig. 1B). The predominant Mt. Konpira land cover is natural vegetation (Fig. 1C). We identified 27 focal trees for ovitraps placement along three transects that covered the altitudinal gradient of Mt. Konpira from 109 to 320 m (Fig. 1C). At each focal tree location, we estimated a series of landscape metrics related to changes in altitude. The metrics include slope, aspect, flow direction, roughness, and terrain roughness index. These altitude-based metrics were derived using a JAXA-based 10-m resolution digital elevation model that uses the Advanced Spaceborne Thermal Emission Reflection Radiometer (ASTER) onboard NASA's Terra platform. Chaves and Moji (2018) describe in detail the interpretation and units of each altitude-related landscape metrics. We estimated the minimum distance from each focal tree to urban land using the land cover classification of Fig. 1C. We also estimated canopy openness (mean and SD) and two ground cover indexes at each sampling location. For ground cover index 1, positive values indicate grounds dominated by leaf-litter, whereas negative values are associated with concrete (Chaves et al., 2015). Ground cover index 2 is related to the type of standing vegetation and was the second principal component of the principal component analysis used to estimate ground cover index 1 (Chaves et al., 2015).

### Mosquito sampling and environmental covariates

Immature mosquitoes were collected from ovitraps (Chaves et al., 2019, 2015), which were sampled biweekly from June 1st, 2014 until June 23rd, 2016. Full details about ovitraps have been presented elsewhere (Chaves et al., 2015; Chaves and Moji, 2018). Briefly, ovitraps were set on May 14th, 2014, and filled with 280 ml of water on that day. Then we let ovitraps (350 ml metallic cans) collect water from rainfall, and natural organic material (*e.g.,* fallen tree-leaves), and recorded the time(s) when ovitraps were dry during our sampling. Twenty-seven ovitraps (*i.e.,* one per focal tree) were uniformly hung at 1.2 m above the ground. From the ovitraps, we removed all pupae and fourth instar mosquito larvae different from *Triperoides bambusa* (Yamada), and then raised the larvae and pupae into adults in order to accurately separate *Ae. albopictus* from *Ae. flavopictus* using morphological characters (Tanaka et al., 1979).

Daily weather data from the Nagasaki city weather station (WMO Station ID: 47,817, available at the Japanese Meteorological Agency website, http://www.data.jma.go.jp/obd/stats/etrn/index.php), located within a 5 km radius from the study site (Fig. 1B), were used to generate biweekly time series of average, SD, and kurtosis of maximum, mean, and minimum temperature and relative humidity. We also generated a time series of biweekly cumulative rainfall in addition to SD and kurtosis time series for daily rainfall over biweekly periods. We measured ovitrap water temperature at the time of sampling with an AD-5617WP infrared thermometer (A&D Co., Tokyo, Japan). From May 2014 to June 2016, we used biweekly images of NDVI and EVI with a 250-m resolution (MOD13Q1) based on MODIS images aboard NASA's Terra satellite (Didan, 2015). We chose NDVI and EVI as reliable indexes associated with vegetation growth (Pettorelli et al., 2005). We also used biweekly Landsat 8, a NASA and USGS collaboration, images that have a 30-m resolution for the same period. The Landsat 8 bands 2 (Blue), 4 (Red), and 5 (Near-Infrared) were corrected using the dark object subtraction 1 (DOS1) algorithm (Chavez, 1996) to correct for shadow-induced reflectance and radiance errors affecting NDVI and EVI estimates. Subsequently, the NDVI was estimated according to the following formula (Vermote et al., 2016):

(1) NDVI=(band5−band4)/(band5+band4)

And using bands 2, 4 and 5 EVI was estimated according to the following formula (Vermote et al., 2016):

(2) EVI=(band5−band4)/(band5+6*band4−7.5*band2+1)

The MODIS and Landsat 8 images were retrieved from https://lpdaac.usgs.gov, maintained by the NASA EOSDIS Land Processes Distributed Active Archive Center (LP DAAC) at the USGS Earth Resources Observation and Science (EROS) Center, Sioux Falls, South Dakota. The MOD13Q1 images were downloaded using the MODIStsp package for the software R (Busetto and Ranghetti, 2016). Landsat 8 images were downloaded, and DOS1 corrections were done, using the SCP plugin in QGIS (Congedo, 2016). The resulting images for the MODIS and Landsat 8 based NDVI and EVI were clipped (Brunsdon and Comber, 2015) to the study area and values extracted for pixels including the location of each one of the 27 focal trees where ovitraps were placed. Using these 27 values we estimated the mean, SD and kurtosis of MODIS and Landsat 8 based NDVI and EVI time series, and also for the ovitrap water temperature measurements. NDVI and EVI time series were smoothed using the loess algorithm (Cleveland and Devlin, 1988), with a smoothing span of 20% of the data and one degree of freedom. The span was selected because 40% of the NDVI and EVI images (21 out of 51) were discarded due to low quality Landsat 8 images that were dominated by cloud cover.

We estimated SD and kurtosis of the environmental covariates as these increasingly recognized higher-order moments can influence the population dynamics of living organisms (Chaves, 2016; Chaves et al., 2011; Chaves and Moji, 2018, 2012; Poh et al., 2019). This was done in accordance with Schmalhausen`s law, which states that populations follow mean environmental values and their patterns of variability (Chaves, 2017a; Chaves and Koenraadt, 2010).

### Spatial analysis

The spatial abundance of *Ae. albopictus*, which was absent from most sampling locations, was studied using zero-inflated count models, fitted by maximum likelihood (Zeileis et al., 2008). We chose this method to account for the zero inflation that emerged from the absence of *Ae. albopictus* from 21 of the 27 ovitraps sampled. For *Ae. flavopictus,* a negative binomial (NB) model was able to capture the over-dispersed (Faraway, 2006; Venables and Ripley, 2002) count nature of its spatial abundance. We then proceeded with a process of model development and selection for each species, where we compared models with sets of highly correlated similar covariates, in order to avoid collinearity issues (Chaves et al., 2019). For *Ae. albopictus,* we selected the four variables with the highest correlation associated with *Ae. albopictus* abundance, which included: *Ae. flavopictus* abundance, SD of canopy openness, altitude, and kurtosis of water temperature. We were not able to test more covariates at the same time given that for the count part of the model, there were only five degrees of freedom. Degrees of freedom were constrained because *Ae. albopictus* was only found at six ovitrap locations. Then we compared zero inflated Poisson models with zero inflated negative binomial models, where models alternatively included the closest distance to urban land or altitude as covariates for the zero inflation. Model selection was based on the minimization of the Akaike Information Criterion (AIC) a metric for model comparison that weights the trade-off between goodness of fit (model likelihood) and parameter number, and chooses the best model, among a set of competing models, often considering highly correlated (a.k.a., colinear) variables, by selecting the model that minimizes AIC (Kuhn and Johnson, 2013). A model with a Poisson distribution for the counts and nearest distance to urban land was selected as best (Table S1). *Aedes flavopictus* was present, at least once, at all 27 ovitrap sampling locations. For *Aedes flavopictus*, the model selection strategy began by comparing a set of 96 models that included the following set of covariates: ground index 1, ground index 2, topographic position index, SD of ovitrap temperature, kurtosis of ovitrap temperature, SD of EVI, SD of NDVI, kurtosis of EVI, and kurtosis of NDVI (Table S2). The models alternatively contained aspect or flow direction, slope or terrain roughness index or roughness, mean or SD canopy openness, average NDVI or EVI, altitude or closest distance to urban land or mean temperature or median temperature (Table S2). In this first stage the best model was one that included the following covariates: *Aedes albopictus* abundance, ground cover index 1 (first principal component), ground cover index 2 (second principal component), aspect, roughness, topographic position index, SD of canopy openness, distance to urban land, SD of temperature, kurtosis of temperature, kurtosis of NDVI, SD of NDVI, average of EVI, SD of EVI, kurtosis of EVI. After six rounds of model selection using backward elimination (Table S3), the “best” negative binomial spatial model covariates included *Aedes albopictus* abundance, aspect, roughness, SD of canopy openness, distance to urban land, SD of temperature, kurtosis of temperature, average of EVI, and kurtosis of EVI. Given the spatial nature of the analyses presented in this section, we estimated Moran's I spatial autocorrelation index on residuals from the best model. Moran`s I is a statistic that has the null hypothesis of spatial independence (Brunsdon and Comber, 2015). The spatial weights matrix used for the estimation of Moran's I index was generated by the identification of all sampling locations within a distance of 66 m for each of the 27 ovitrap locations in our study. The 66 m distance radius was chosen because this was the largest minimum distance between any two sampling locations. Once neighbors were identified, weights were made proportional to the number of neighbors for each sampling location (Brunsdon and Comber, 2015).

### Temporal analysis

Temporal patterns were studied using formal methods for time series analysis (Shumway and Stoffer, 2011). The analysis began with the estimation a “null” autoregressive model for each mosquito abundance time series. The lags were selected based on the autocorrelation and partial autocorrelation functions. The null autoregressive models were then used to pre-whiten the time series of environmental covariates and estimate cross-correlation functions between the residuals of the null autoregressive models and the pre-whitened time series of the environmental covariates (Chaves, 2017b). This process allowed the selection of lags for covariates, which do not spuriously emerge by the response and predictor time series having a similar autocorrelation structure (Hoshi et al., 2014). The selected covariates and lags, which considered the highest correlations for each covariate up to three biweeks of lag (Hoshi et al., 2017), were then included in “full” zero-inflated count models that alternatively had Poisson and negative binomial count distributions (Tables S4 and S5) and best “full” model was selected by AIC minimization (Shumway and Stoffer, 2011). The best “full” models were simplified using AIC-based backward elimination (Kuhn and Johnson, 2013). We employed zero-inflated count models to account for the excess abundance of zeroes that do not correspond to a count distribution (Korner-Nievergelt et al., 2015). Zero-inflated models assume that observed zeroes represent a mixture of zeroes from a count distribution, *e.g.,* Poisson or negative binomial, and structural zeroes that could reflect unsuitable conditions for the dynamics of a process (MacDonald and Zucchini, 1997). In the models, abundance was weighted by the number of ovitraps with water, provided some traps became naturally dry during the study. On the 46th and 51st biweeks, three traps were lost after an unusual snowstorm and around half of the remaining traps (11 of 24) were vandalized, respectively (Chaves and Moji, 2018).

Using information from the zero-inflated part of the models, we estimated the water temperature threshold for which the probability of the model having a non-structural zero is 50% (WTp50) by solving the following equation:

(3) Intercept+Coefficient_Water_Temperature*WTp50=0

Briefly, that means that below WTp50 a zero likely represents adverse conditions for the presence of pupae or fourth instar larvae of the studied mosquitoes, while a zero above WTp50 is more likely part of the population dynamics of the studied mosquito species (Chaves and Moji, 2018).

### Data availability

Environmental data came from public sources cited within the manuscript, all other data are presented within the manuscript and raw data to reproduce figures are in the supplementary material.

## Results

**Fig. 2:**
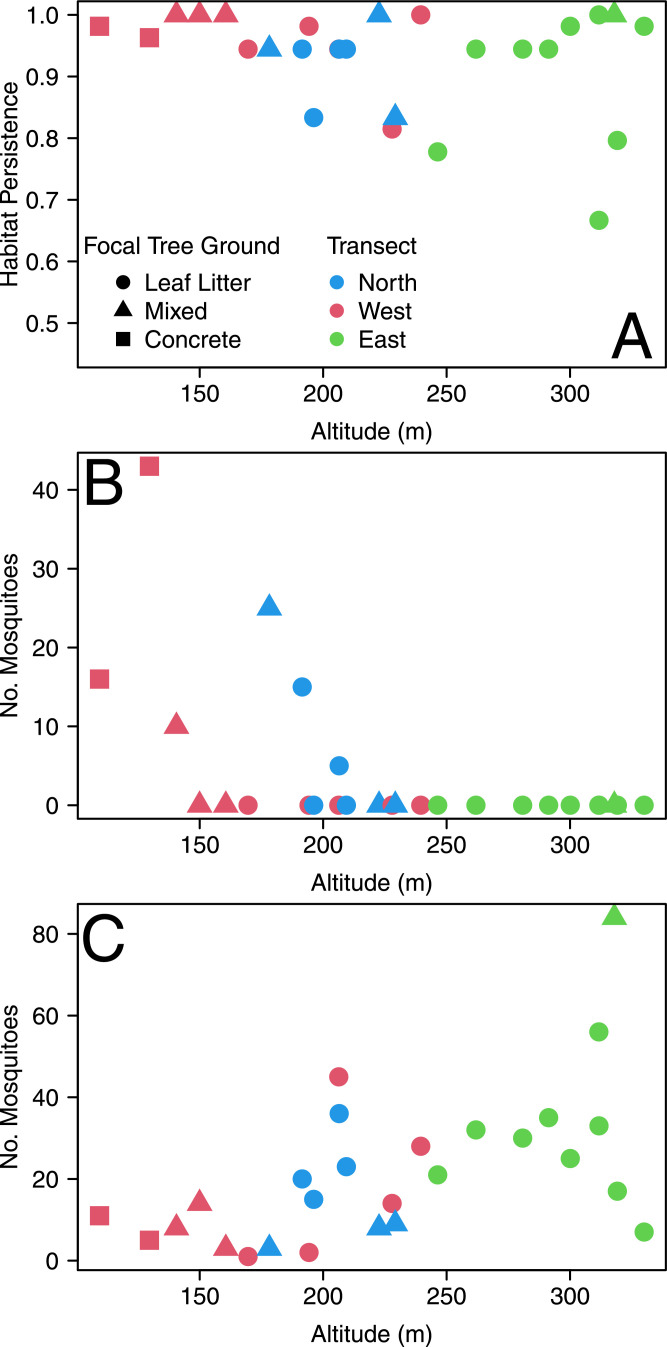
Spatial mosquito abundance patterns in 2014–2016 at Mt. Konpira, Nagasaki, Japan (A) Habitat persistence, this plot presents the proportion of times an ovitrap had water as function of elevation (B) *Aedes albopictus* total abundance during our study period as function of elevation (C) *Aedes flavopictus* total abundance during our study period as function of elevation. In panels B and C symbol and color indicate, respectively, dominant ground and transect, for details please refer to the inset legend of panels A.

During the study period, we collected a total of 114 *Ae. albopictus* and 585 *Ae. flavopictus* 4th instar larvae and pupae. For *Ae. albopictus,* we collected 17 pupae, while for *Ae. flavopictus* we collected 52 pupae. During the study, all ovitraps had water over 50% of the times we sampled mosquitoes, and ovitraps at higher elevations were more likely to get dry (Fig. 2A). *Ae. albopictus* was found only below 210 m (Fig. 2B), but *Ae. flavopictus* was present all over Mt. Konpira altitudinal gradient, increasing its abundance with altitude (Fig. 2C). Parameter estimates for the best model explaining the spatial abundance of *Ae. albopictus* are presented in Table 1 and for *Ae. flavopictus* are presented in Table 2. Both models had not significant Moran`s I indexes for the residuals (Tables 1 and 2) indicating that inferences are valid since the error was spatially independent. Among factors unique to the abundance of each species, we observed that *Ae. albopictus* increased its abundance with altitude (2.7% per m of increase) and mainly with the kurtosis of ovitrap water temperature (278 times per unit of increase in kurtosis). In contrast, *Ae. flavopictus* increased its abundance with landscape aspect, *i.e.,* the direction of the slope measured in degrees (Wilson et al., 2007), indicating this mosquito increased its abundance westward by 0.26% per degree of aspect increase (Table 2); with terrain roughness, *i.e.,* the maximum elevation difference between the set of nine cells composed by the focal cell containing an ovitrap and its eight surrounding neighbors (Wilson et al., 2007), increasing its abundance by 7.3% per m of roughness increase (Table 2). *Ae. flavopictus* was positively associated with mean EVI, increasing its abundance by 16.5% per unit of EVI increase, and negatively with the kurtosis of EVI, decreasing its abundance by 35.9% per unit of kurtosis increase (Table 2). *Ae. flavopictus* was negatively associated with increased variability in temperature, decreasing its abundance by 91.2% per unit of SD increase (Table 2).

**Table 1:** Parameter estimates for the best zero inflated Poisson spatial model explaining the spatial abundance of *Aedes albopictus* along Mt. Konpira, Nagasaki, Japan. Abundance Change is per unit increase (UI) in the covariate. The inference for parameter estimates is based on: Z Wald Tests for the count model coefficients and a 1000 replicates Monte Carlo randomization for the Moran's I index of the model residuals. Units indicate the measurement units for each covariate considered for parameter estimation. S.E. stands standard error.

Count model with log link Parameter (units)	Abundance Change	Estimate	S.E.	z value	Pr(>|z|)
Intercept	–	−15.547	6.759	−2.300	2.14E-02*
*Aedes flavopictus* abundance (Individuals/trap)	0.926	−0.077	0.020	−3.900	9.62E-05*
SD canopy openness (%)	1.037	0.036	0.010	3.762	0.000169*
Kurtosis of the ovitrap water temperature ( °C)	277.841	5.627	2.685	2.096	0.036076*
Altitude (m)	1.027	0.027	0.011	2.361	0.01821*
Moran's I	–	0.259	–	–	0.057
Zero-inflation model coefficients (binomial with logit link)					
Parameter	Estimate	S.E.	z value	Pr(>|z|)	
Intercept		−1.583	1.092	−1.449	0.1473
Nearest distance to urban land (m)	0.033	0.016	2.149	0.0317*	

⁎ Statistically significant (*P* < 0.05).

**Table 2:** Parameter estimates for the best negative binomial spatial model explaining the spatial abundance of *Aedes flavopictus* along Mt. Konpira, Nagasaki, Japan. Abundance Change is per unit increase (UI) in the covariate. The inference for parameter estimates is based on: Z Wald Tests for the count model coefficients and a 1000 replicates Monte Carlo randomization for the Moran's I index of the model residuals. Units indicate the measurement units for each covariate considered for parameter estimation. S.E. stands for standard error.

Parameter (units)	Proportional Abundance	Estimate	S. E.	z value	Pr(>|z|)
	Change				
Intercept	–	12.117	3.484	3.478	0.000505*
*Aedes albopictus* abundance (Individuals/trap)	1.057	0.056	0.019	2.921	0.003489*
Aspect (degrees)	1.002	0.002	0.001	2.665	0.007688*
Roughness (m)	1.073	0.071	0.015	4.766	1.88E-06*
SD Canopy openness (%)	0.930	−0.072	0.029	−2.447	0.014409*
Distance to Urban Land (m)	1.006	0.006	0.001	6.266	3.71E-10*
SD Temperature (°C)	0.088	−2.435	0.540	−4.51	6.49E-06*
EVI (adimensional ratio)	1.165	0.153	0.053	2.896	0.003783*
Kurtosis of EVI (adimensional ratio)	0.641	−0.445	0.208	−2.138	0.03249*
Overdispersion	–	6.680	2.700	–	–
Moran's I	–	0.078	–	–	0.268

⁎ Statistically significant (*P* < 0.05).

**Fig. 3:**
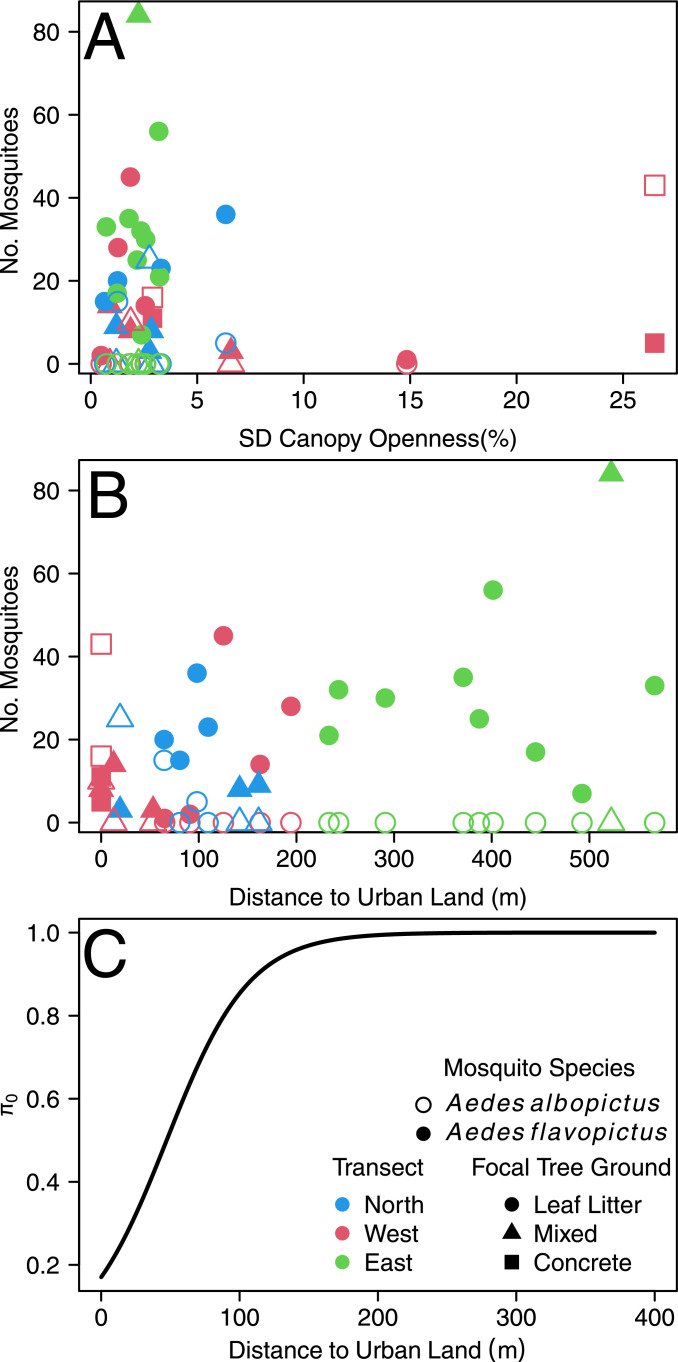
Mosquito abundance as function of selected spatial variables in 2014–2016 at Mt. Konpira, Nagasaki, Japan (A) SD Canopy Openness (B) Minimum distance to urban land (C) Zero inflation probability function for *Aedes albopictus*. The y axis shows the probability of structural 0 ($\pi_{0}$) from Table 1, where the probability increases following a sigmoid function of the distance to urban land (x axis). In panels A and B symbols and colors follow the ones presented in the inset legend of panel C, with hollow symbols representing *Aedes albopictus*and filled symbols representing *Aedes flavopictus.*

Regarding common significant factors for the spatial abundance of both species, parameter estimates show that each additional *Ae. flavopictus* individual decreased *Ae. albopictus* abundance by 7.4% (Table 1), while *Ae. flavopictus* abundance increased by 5.7% for each additional *Ae. albopictus* individual (Table 2). Increments in SD of canopy openness (Fig. 3A) had a negative impact on *Ae. flavopictus,* decreasing its abundance by 7.0% per unit of SD of % canopy openness (Table 2), while this variable was positively associated with *Ae. albopictus*, increasing its abundance by 3.7% per unit of SD of % canopy openness (Table 1). The association with minimum distance to urban land was also contrasting (Fig. 3B), with *Aedes albopictus* decreasing (Table 1), and *Aedes flavopictus* increasing (0.6% by m, Table 2), its abundance as such distance increased. Indeed, for *Ae. albopictus*, the zero inflation function shows that the probability of occurrence was null for distances over 200 m from urban land (Fig. 3C).

**Fig. 4:**
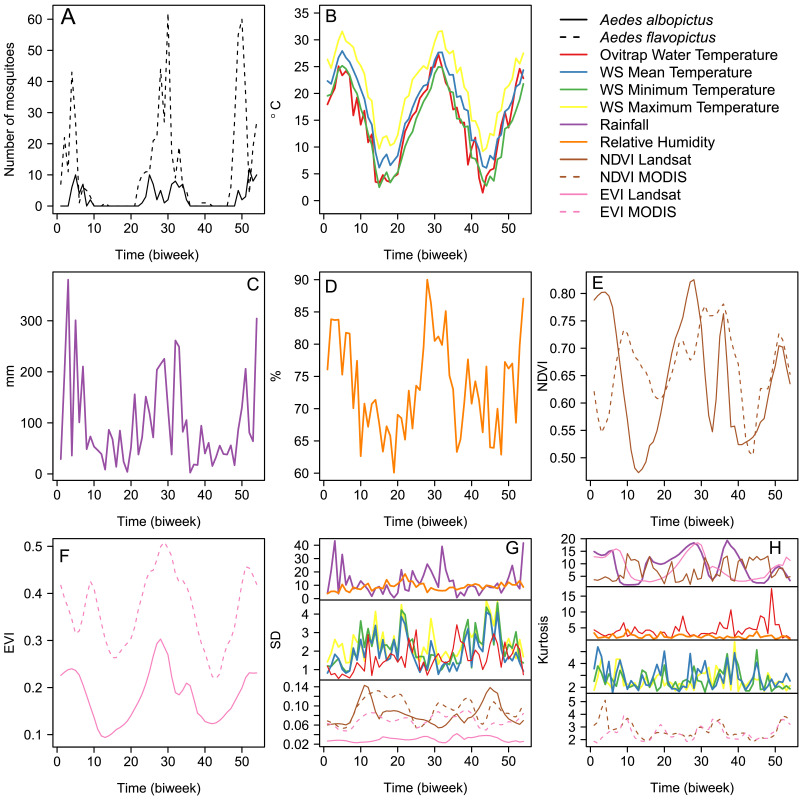
Time Series data (A) Total biweekly mosquito abundance, 4th instar larvae and pupae (B) Temperature, where WS stands for Weather Station (C) Biweekly cumulative Rainfall (D) Relative Humidity (E) Landsat 8 and MODIS based NDVI (F) Landsat 8 and MODIS based EVI (G) SD of environmental covariates (H) Kurtosis of environmental covariates. For details about time series coding please refer to the inset legend. In panels G and H time series were stacked in subplots according to their maximum and minimum value to ease the visualization of each time series, thus each subplot has a different scale, the top panels having the widest scale, while the bottom panels have the narrowest scale.

Temporal mosquito abundance patterns were seasonal, with *Ae. flavopictus* having an extended pre-imaginal season when compared with *Ae. albopictus* (Fig. 4A). For example, *Ae. albopictus* was continuously absent from ovitraps from biweeks 10 to 22 and 36 to 48, whereas *Ae. flavopictus* was only continuously absent from biweeks 14 to 21 and 42 to 46. The seasonal pattern followed changes observed in air and ovitrap water temperature (Fig. 4B), where *Ae. albopictus* was only present when the temperature was within double-digit ranges, unlike *Ae. flavopictus* that was present at single-digit temperatures. Similar patterns were observed for rainfall (Fig. 4C), relative humidity (Fig. 4D), NDVI (Fig. 4E) and EVI (Fig. 4F), where the two mosquito species were present when all these environmental variables had values above their average. The largest SD was observed for rainfall and relative humidity, without a clearly marked seasonal pattern, followed by the SD of the temperature time series and the Landsat 8 and MODIS based vegetation indices (Fig. 4G). Meanwhile, kurtosis was largest for rainfall, Landsat 8 based NDVI and EVI (Fig. 4H). Ovitrap water temperature, increased its kurtosis at the end of the study period, being more leptokurtic, *i.e.*, with more kurtosis (Fig. 4H). All other variables were more platykurtic, *i.e.*, with low kurtosis, the air temperature variables having similar patterns among themselves, but different from the MODIS based NDVI and EVI (Fig. 4H).

**Fig. 5:**
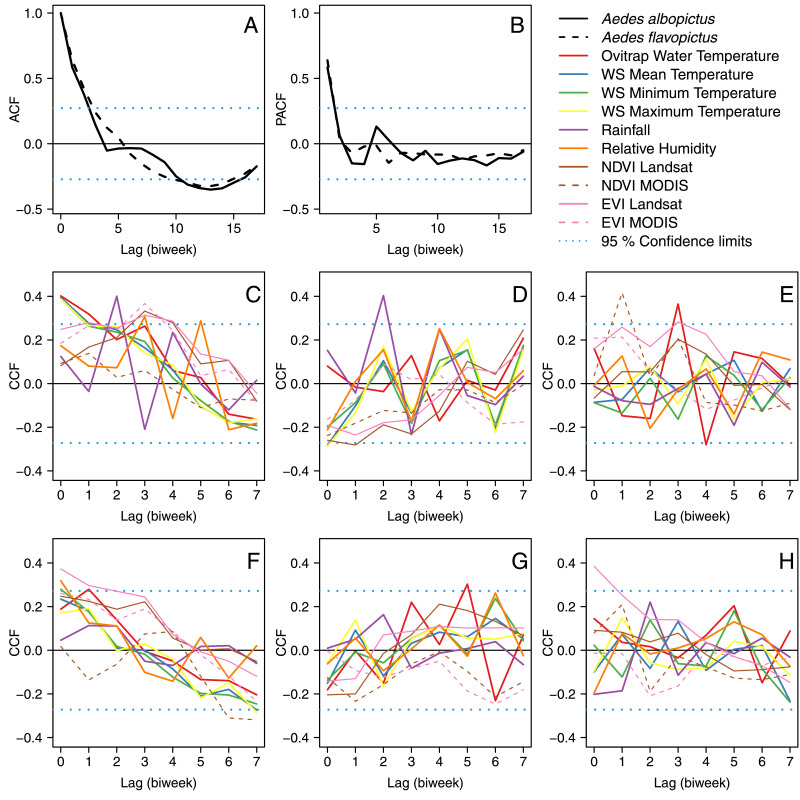
Correlation Functions (A) Autocorrelation Function, ACF of mosquito abundance, 4th instar larvae and pupae (B) Partial ACF, PACF of mosquito abundance (C) Cross Correlation Function, CCF between *Aedes albopictus* abundance and mean of environmental covariates (D) CCF between *Ae albopictus* and SD of environmental covariates (E) CCF between *Ae albopictus* and kurtosis of environmental covariates (F) CCF between *Aedes flavopictus* abundance and mean of environmental covariates (G) CCF between *Ae flavopictus* and SD of environmental covariates (H) CCF between *Ae flavopictus* and kurtosis of environmental covariates. Variables are color coded as presented in the inset legend of the figure. In all panels dotted blue lines indicate the 95 confidence limits inside which correlations are expected by chance. This means that peaks outside the blue lines are the time lags at which mosquito abundance was associated with itself in the ACF and PACF plots, or with the covariates in the CCF plots. (For interpretation of the references to color in this figure legend, the reader is referred to the web version of this article.)

Both the autocorrelation function (Fig. 5A) and partial autocorrelation function (Fig. 5B) were first-order autoregressive (*i.e.,* significantly associated at 1 time lag) for both mosquito species and were used to fit first-order autoregressive models. The cross-correlation functions of *Ae. albopictus* temporal abundance with mean (Fig. 5C), SD (Fig. 5D), and kurtosis (Fig. 5E) of the environmental covariates suggested *Ae. albopictus* abundance was associated with water and air temperature, rainfall, Landsat-based EVI and NDVI, and MODIS-based NDVI at lags presented in Table S4, where we also tested models with highly correlated variables separately (Chaves et al., 2019). The cross-correlation functions of *Ae. flavopictus* temporal abundance with mean (Fig. 5F), SD (Fig. 5G), and kurtosis (Fig. 5H) of the environmental covariates suggested *Ae. flavopictus* abundance was associated with relative humidity and Landsat based EVI at lags presented in Table S5. Model selection for the best full zero-inflated count model for *Ae. albopictus* and *Ae. flavopictus* are, respectively, presented in Tables S6 and S7, and both models included ovitrap water temperature as a covariate for zero inflation.

Both SD and kurtosis of the environmental covariates were associated with mosquito abundance, as revealed by the cross-correlation analysis (Fig. 2). The best model for explaining the abundance of *Ae. albopictus* (Table 3) had a two weeks autoregressive component. *Ae. albopictus* abundance increased 11.10% per unit increase of ovitrap water temperature kurtosis, and by 45.00% per unit increase of MODIS based NDVI kurtosis, both variables having their impacts with a six weeks lag. For *Ae. flavopictus*, the best model (Table 3) had a positive two weeks autoregressive component and increased 17.4% for each EVI unit increase with six weeks of lag. Based on parameters from the zero inflation functions presented in Table 3, we estimated WTp50 (mean ± SE) for *Ae. albopictus* 15.64 ± 11.38 °C and *Ae. flavopictus* 6.32 ± 18.96 °C.

**Table 3:** Parameter estimates for the best zero inflated Poisson time series count model explaining the temporal abundance of *Aedes albopictus* and the best zero inflated negative binomial time series count model explaining the temporal abundance of *Aedes flavopictus* during 2014–2016 at Mt. Konpira, Nagasaki, Japan. Lag indicates the covariate time lag measured in biweeks. Abundance Change are per unit increase (UI) in the covariate, where NDVI and EVI are adimensional ratios, while Temperature was measured in °C. S.E. stands for standard error. The inference for parameter estimates is based on: Z Wald Tests for the count model coefficients, a $\chi^{2}$likelihood ratio test for the zero inflation (whose degrees of freedom are indicated by df).

Species	Count model, log link Parameter (Lag)	Abundance Change	Estimate	S.E.	z value	Pr(>|z|)
*Aedes*	Intercept	—	−3.448	0.368	−9.380	1< 2e-16*
*albopictus*	Auto-regressive (1)	1.068	0.066	0.026	2.537	0.011190*
	Kurtosis of water temperature (3)	1.111	0.105	0.022	4.736	2.18e-06
	Kurtosis of MODIS based NDVI (3)	1.450	0.371	0.099	3.740	0.000184
	Zero-inflation model coefficients, binomial with logit link					
	Parameter (Lag)		Estimate	S.E.	$\chi^{2}\left(\text{df}\right)$	Pr(>|$\chi^{2}$|)
	Intercept		11.951	4.371	20.451 (1)	0.000006*
	Water temperature (0)	−0.764	0.277	23.670 (1)	0.000001*	
Species	Count model, log link Parameter (Lag)	Abundance Change	Estimate	S.E.	z value	Pr(>|z|)
*Aedes*	Intercept	—	−4.413	0.862	−5.122	3.03e-07*
*flavopictus*	Auto-regressive (1)	1.027	0.027	0.011	2.355	0.0185*
	Landsat based EVI (3)	1.174	0.161	0.044	3.618	0.000297*
	Overdispersion	—	0.988	0.260	—	—
	Zero-inflation model coefficients, binomial with logit link					
	Parameter (Lag)		Estimate	S.E.	$\chi^{2}\left(\text{df}\right)$	Pr(>|$\chi^{2}$|)
	Intercept		98.880	213.050	14.226 (1)	0.000162*
	Water temperature (0)	−15.650	33.840	19.957 (1)	7.918e-06*	

*Statistically significant (*P* < 0.05).

## Discussion

In this study, we found evidence suggesting the existence of a species limiting similarity driving *Ae. flavopictus* and *Ae. albopictus* co-occurrence patterns at Mt. Konpira, Nagasaki, Japan*.* Our data suggest that co-existence between these two species might be partially shaped by a different response to environmental conditions within a gradient, with differences driven by different associations between the spatial and temporal abundance of these two species with common environmental variables. For example, our spatial analysis clearly showed that *Ae. albopictus* was more abundant near urban land use, in opposition to what was observed for *Ae. flavopictus* whose abundance increased farther away from urban land use, establishing that the gradient of distance to urban land use was key for the abundance patterns of both species along the altitudinal gradient of Mt. Konpira.

However, differences in the response of *Ae. albopictus* and *Ae. flavopictus* to environmental factors are unlikely only due to preferences along fixed mean values in the environmental gradient, but also to the environmental variability pattern along the gradient (Levins, 1968), an expectation from Schmalhausen's law. For example, *Ae. albopictus* was more abundant in locations with high kurtosis (leptokurtic distributions) in ovitrap water temperature. Leptokurtic distributions are less variable around the mean than the extremes (Ross, 2014), suggesting *Ae. albopictus* colonizes locations that undergo more extreme temperature changes, something that is associated with places with a high SD in canopy cover, which are more sensitive to temperature variation through sunlight exposure (Colinet et al., 2015; Service, 1965). By contrast, *Ae flavopictus* decreased its abundance with the SD of canopy cover, with the SD of ovitrap water temperature and the kurtosis of EVI, suggesting this species preferred less variable environments and platykurtic environments, *i.e.,* with more variability around the mean than in the extremes of the distribution (Ross, 2014), a possibility further reinforced by the increase of abundance positive association with average values of EVI, and aspect and roughness conditions that could favor shadow generation, and less variable thermal environments (Colinet et al., 2015), at our study site (Chaves et al., 2019; Chaves and Moji, 2018). These differences, in response to landscape variables, on the abundance of these mosquitoes more clearly articulate why urban land use might drive differences in the abundance of *Ae. albopictus* and *Ae. flavopictus*, following a pattern similar to what we have observed for both species ovitrap colonization (Chaves et al., 2020).

Temporal abundance patterns also suggest both species have a different response to changing thermal environments. For example, there was a difference of more than 9 °C in the threshold temperature for structural zeros (WTp50) in the zero-inflation part of the temporal models, with *Ae. flavopictus* likely to be present above 6.3 °C. This reduction in pre-imaginal absence from ovitraps can be explained by an unusually warm winter during 2015–2016 (Chaves et al., 2018), which lasted only five biweeks as compared to the eight biweeks of pre-imaginal absence from ovitraps during the 2014–2015 winter. By contrast, *Ae. albopictus* was absent for 12 biweeks during the two winters of this study, something likely to reflect its WTp50 near 15 °C. The higher WTp50 of *Ae. albopictus* supports the proposal that *Ae. albopictus* colonization of northern Japan (Kobayashi et al., 2002; Maekawa et al., 2016b; Mogi and Tuno, 2014) and elsewhere (Mogi et al., 2015) might be partially driven by global warming. The relationship of both species with temperature might also be related to their overwintering strategy through egg diapause, which is known to be shorter for *Ae. flavopictus* than *Ae. albopictus* in warm subtropical environments (Mogi, 1996), where *Ae. flavopictus* might be more sensitive to temperature cues that can stop egg diapause, a mechanism not common in *Ae. albopictus* where photoperiod has been proposed as the dominant regulatory cue for egg diapause (Armbruster, 2016), something we observed in the field in Nagasaki (Chaves et al., 2018), but not for *Ae. albopictus* eggs from mosquitoes living under daylight-lengths similar to those of tropical environments (Tsunoda et al., 2015).

*Ae. albopictus* temporal abundance also increased as temporal changes in temperature and remotely sensed vegetation growth became more leptokurtic, *i.e.,* with high kurtosis, meaning that temperature is relatively more variable towards the extremes of the distribution than around its mean (Ross, 2014). This phenomenon is similar to what we have observed for *Aedes (Finlaya) japonicus* (Theobald) at this study site (Chaves and Moji, 2018). In contrast, *Ae. flavopictus* abundance increased with vegetation growth, something re-inforcing its preference for locations far from urban land use and that are constrained to have low variability given the bounded nature of remotely sensed vegetation growth indexes (Pettorelli et al., 2005).

Finally, in a world undergoing global-scale environmental change, mosquitoes seem to be one of the many medically important taxa seeing unexpected changes in their ecology (Chaves, 2017a) and *Ae. albopictus* and *Ae. flavopictus* seem to be no exception. Here, it is worth to start by highlighting that other common mosquito species, at the larval stage, at the study site unlikely interact with *Ae. albopictus* and *Ae. flavopictus*, as we have quantitatively measured for *Ae. japonicus* at the study site (Chaves and Moji, 2018). For *Tr. bambusa*, the most abundant and ubiquitous species at Mt. Konpira (Tsuda et al., 1994; Zea Iriarte et al., 1991), we also have recorded a similar pattern of co-occurrence with *Ae. albopictus* and *Ae. flavopictus*, suggesting a lack of differential interaction at the study site (Chaves et al., 2019). Other species, for example *Armigeres subalbatus* (Coquillet), were very rarely sampled with the ovitraps (Chaves et al., 2015). The emergence of *Ae. flavopictus* as a dominant mosquito in Mt. Konpira (Chaves, 2016), occurs in an area where it was not recorded around 30 years ago (Tsuda et al., 2003, 1994; Zea Iriarte et al., 1991). Seventy years ago this species was one of the rarest at the larger biogeographical scale of Nagasaki prefecture (Omori et al., 1952). Moreover, the recent interception of *Ae. flavopictus* in Europe (Ibáñez-Justicia et al., 2019) and its pattern of co-occurrence and potential interaction with two major globally invasive species, *Ae. albopictus* and *Ae. japonicus* in their native range (Chaves, 2016) deserves further attention. Particularly, as this study shows that distance to urban land use is key for the co-occurrence of *Ae. albopictus* and *Ae. flavopictus,* with *Ae. flavopictus* also more likely to be present as larvae at lower temperatures than *Ae. albopictus*. These results highlight the need to keep studying *Ae. flavopictus* in its native range (Chaves, 2017b), but also for monitoring its potential invasion as already done in Europe and Australia (Doggett et al., 2019; Ibáñez-Justicia et al., 2019). Particular attention should be given to monitoring larvae, since *Ae. flavopictus* seems scarce when sampled with CO_2_ baited adult light traps, despite having high landing rates when sampled at the same sites where deployed CO_2_ baited light traps fail to successfully catch large quantities of adult *Ae. flavopictus* mosquitoes (Maekawa et al., 2016b). The nature of interspecific interactions between *Ae. albopictus* and *Ae. flavopictus* also requires further research, since spatially our results showed that *Ae. flavopictus* had a negative association with the pre-imaginal abundance of *Ae. albopictus,* and our previous efforts with mechanistic and statistical models, developed with adult mosquito data (Chaves, 2016), suggested both species interact antagonistically.

## Author contributions

LFC Conceptualization, LFC Formal analysis, LFC & MDF Investigation, LFC Data curation, LFC Funding acquisition, LFC Project administration, LFC & MDF Methodology, LFC Software, LFC Supervision, LFC Validation, LFC Visualization, LFC Writing - original draft, LFC & MDF Writing - review & editing & approving final version.

## Funding

This research was funded by 10.13039/100008608Sumitomo Foundation grant no. 153107 to LFC. The work by MDF is supported by an appointment to the NASA Postdoctoral Program at the NASA Goddard Space Flight Center, administered by Universities Space Research Association under contract with NASA.

## Compliance with ethical standards

Ethical approval: this research did not include human subjects. Nagasaki City direction of green areas (Midori no Ka) kindly provided all relevant permits to perform the study.

## Declaration of Competing Interest

The authors declare that they have no known competing financial interests or personal relationships that could have appeared to influence the work reported in this paper.

## Appendix Supplementary materials

Image, application 1

Image, application 1
