# Supplementary material for: Aedes albopictus and Aedes flavopictus (Diptera: Culicidae) pre-imaginal abundance patterns are associated with different environmental factors along an altitudinal gradient
Source: Curr Res Insect Sci. 2020 Oct 15;1:100001. doi: 10.1016/j.cris.2020.100001 (PMC9387439; doi:10.1016/j.cris.2020.100001)
Supplement: Supplementary file 1 [file mmc1.pdf]

Table S1 Model Selection for the best zero inflated spatial model for the abundance of *Aedes albopictus* 4th instar larvae and pupae in Mt. Konpira, Nagasaki – Japan. Count indicates covariates considered for modeling spatial counts, while Zero inflation indicates covariates considered for modeling the zero inflation. The covariates considered were selected based on their correlation with Aedes albopictus spatial abundance. AIC indicates the Akaike Information Criterion, which is **bolded** for the best model, i.e., the one minimizing AIC, while best models at each single step are both ***bolded and italicized***. All models were weighted by the number of times traps had water when sampled.

Round	Count	Zero inflation	Distribution	AIC
1	*Aedes flavopictus* abundance, SD of canopy openness, elevation, kurtosis of water temperature	Distance to urban land	Negative binomial	58.02
	Aedes flavopictus abundance, SD of canopy openness, elevation, kurtosis of water temperature	Elevation	Negative binomial	60.95
	Aedes flavopictus abundance, SD of canopy openness, elevation, kurtosis of water temperature	Distance to urban land	Poisson	***56.02***
	Aedes flavopictus abundance, SD of canopy openness, elevation, kurtosis of water temperature	Elevation	Poisson	58.95

Table S2 Model Selection for the best “full” negative binomial spatial model explaining the cumulative spatial abundance of *Aedes flavopictus* 4th instar larvae and pupae in Mt. Konpira, Nagasaki – Japan. Fixed covariates indicate covariates that were considered in all models, while Changing covariates indicate covariates that were changed because they presented a high correlation (over 80%). AIC indicates the Akaike Information Criterion, which is **bolded** for the best “Full” model, i.e., the one minimizing AIC.

Fixed Covariates	Changing Covariates	AIC
Ground Index 1, Ground index 2 , TPI (Topographic position index), SD of Temperature, Kurtosis of the temperature, SD EVI, SD NDVI, Kurtosis of EVI, Kurtosis of NDVI	Aspect, Slope, MCO (Mean Canopy Openess), Elevation, Average NDVI	218.07
	FD (Flow direction), Slope, MCO, Elevation, Average NDVI	223.74
	Aspect, TRI (Terrain Roughness Index), MCO, Elevation, Average NDVI	218.05
	FD, TRI, Slope, MCO, Elevation, Average NDVI	223.75
	Aspect, Roughness, MCO, Elevation, Average NDVI	218.02
	FD, Roughness, Slope, MCO, Elevation, Average NDVI	223.39
	Aspect, Slope, SDCO (SD Canopy Openess), Elevation, Average NDVI	217.21
	FD, Slope, SDCO, Elevation, Average NDVI	222.67
	Aspect, TRI, SDCO, Elevation, Average NDVI	217.39
	FD, TRI, Slope, SDCO, Elevation, Average NDVI	222.83
	Aspect, Roughness, SDCO, Elevation, Average NDVI	216.69
	FD, Roughness, Slope, SDCO, Elevation, Average NDVI	221.58
	Aspect, Slope, MCO, Elevation, Average EVI	217.48
	FD, Slope, MCO, Elevation, Average EVI	223.59
	Aspect, TRI, MCO, Elevation, Average EVI	217.80
	FD, TRI, Slope, MCO, Elevation, Average EVI	223.85
	Aspect, Roughness, MCO, Elevation, Average EVI	217.57
	FD, Roughness, Slope, MCO, Elevation, Average EVI	223.30
	Aspect, Slope, SDCO, Elevation, Average EVI	216.36
	FD, Slope, SDCO, Elevation, Average EVI	222.27
	Aspect, TRI, SDCO, Elevation, Average EVI	217.32
	FD, TRI, Slope, SDCO, Elevation, Average EVI	222.96
	Aspect, Roughness, SDCO, Elevation, Average EVI	215.98
	FD, Roughness, Slope, SDCO, Elevation, Average EVI	221.13
	Aspect, Slope, MCO, Distance to Urban Land (DURB), Average NDVI	216.91
	FD, Slope, MCO, DURB, Average NDVI	223.50
	Aspect, TRI, MCO, DURB, Average NDVI	216.99
	FD, TRI, Slope, MCO, DURB, Average NDVI	223.49
	Aspect, Roughness, MCO, DURB, Average NDVI	216.56
	FD, Roughness, Slope, MCO, DURB, Average NDVI	222.87
	Aspect, Slope, SDCO, DURB, Average NDVI	215.88
	FD, Slope, SDCO, DURB, Average NDVI	222.50
	Aspect, TRI, SDCO, DURB, Average NDVI	216.24
	FD, TRI, Slope, SDCO, DURB, Average NDVI	222.66

Aspect, Roughness, SDCO, DURB, Average NDVI	214.76
FD, Roughness, Slope, SDCO, DURB, Average NDVI	220.98
Aspect, Slope, MCO, DURB, Average EVI	216.10
FD, Slope, MCO, DURB, Average EVI	223.31
Aspect, TRI, MCO, DURB, Average EVI	216.70
FD, TRI, Slope, MCO, DURB, Average EVI	223.55
Aspect, Roughness, MCO, DURB, Average EVI	216.09
FD, Roughness, Slope, MCO, DURB, Average EVI	222.80
Aspect, Slope, SDCO, DURB, Average EVI	214.85
FD, Slope, SDCO, DURB, Average EVI	222.14
Aspect, TRI, SDCO, DURB, Average EVI	216.10
FD, TRI, Slope, SDCO, DURB, Average EVI	222.77
**Aspect, Roughness, SDCO, DURB, Average EVI**	**214.05**
FD, Roughness, Slope, SDCO, DURB, Average EVI	220.66
Aspect, Slope, MCO, Mean Temperature (MET), Average NDVI	220.24
FD, Slope, MCO, MET, Average NDVI	225.92
Aspect, TRI, MCO, MET, Average NDVI	219.79
FD, TRI, Slope, MCO, MET, Average NDVI	225.69
Aspect, Roughness, MCO, MET, Average NDVI	219.97
FD, Roughness, Slope, MCO, MET, Average NDVI	226.05
Aspect, Slope, SDCO, MET, Average NDVI	221.20
FD, Slope, SDCO, MET, Average NDVI	226.32
Aspect, TRI, SDCO, MET, Average NDVI	220.18
FD, TRI, Slope, SDCO, MET, Average NDVI	225.78
Aspect, Roughness, SDCO, MET, Average NDVI	221.07
FD, Roughness, Slope, SDCO, MET, Average NDVI	226.47
Aspect, Slope, MCO, MET, Average EVI	220.49
FD, Slope, MCO, MET, Average EVI	226.51
Aspect, TRI, MCO, MET, Average EVI	219.57
FD, TRI, Slope, MCO, MET, Average EVI	226.07
Aspect, Roughness, MCO, MET, Average EVI	219.57
FD, Roughness, Slope, MCO, MET, Average EVI	226.33
Aspect, Slope, SDCO, MET, Average EVI	221.92
FD, Slope, SDCO, MET, Average EVI	227.10
Aspect, TRI, SDCO, MET, Average EVI	220.56
FD, TRI, Slope, SDCO, MET, Average EVI	226.43
Aspect, Roughness, SDCO, MET, Average EVI	221.38
FD, Roughness, Slope, SDCO, MET, Average EVI	227.03
Aspect, Slope, MCO, Median Temperature (MDT), Average NDVI	219.10
FD, Slope, MCO, MDT, Average NDVI	225.44
Aspect, TRI, MCO, MDT, Average NDVI	219.11
FD, TRI, Slope, MCO, MDT, Average NDVI	225.52
Aspect, Roughness, MCO, MDT, Average NDVI	218.81
FD, Roughness, Slope, MCO, MDT, Average NDVI	225.61
Aspect, Slope, SDCO, MDT, Average NDVI	219.57
FD, Slope, SDCO, MDT, Average NDVI	225.56

	Aspect, TRI, SDCO, MDT, Average NDVI	218.87
	FD, TRI, Slope, SDCO, MDT, Average NDVI	225.17
	Aspect, Roughness, SDCO, MDT, Average NDVI	219.46
	FD, Roughness, Slope, SDCO, MDT, Average NDVI	225.85
	Aspect, Slope, MCO, MDT, Average EVI	218.58
	FD, Slope, MCO, MDT, Average EVI	225.51
	Aspect, TRI, MCO, MDT, Average EVI	217.99
	FD, TRI, Slope, MCO, MDT, Average EVI	225.27
	Aspect, Roughness, MCO, MDT, Average EVI	217.48
	FD, Roughness, Slope, MCO, MDT, Average EVI	225.27
	Aspect, Slope, SDCO, MDT, Average EVI	219.33
	FD, Slope, SDCO, MDT, Average EVI	225.75
	Aspect, TRI, SDCO, MDT, Average EVI	218.02
	FD, TRI, Slope, SDCO, MDT, Average EVI	225.06
	Aspect, Roughness, SDCO, MDT, Average EVI	218.54
	FD, Roughness, Slope, SDCO, MDT, Average EVI	225.69

Table S3 Model Selection for the "best" negative binomial spatial model explaining the cumulative spatial abundance of *Aedes flavopictus* 4th instar larvae and pupae in Mt. Konpira, Nagasaki – Japan. AIC indicates the Akaike Information Criterion, which is **bolded** for the "best" model, i.e., the one minimizing AIC.

Round (Removed covariate)	Covariates	AIC
0 (None removed)	*Aedes albopictus* abundance, ground index 1 , ground index 2, Aspect, roughness, TPI (Topographic Position Index), SD canopy openness, distance to urban land , SD Temperature, Kurtosis Temperature, Kurtosis NDVI, SD NDVI, Average EVI, SD EVI, Kurtosis EVI	214.06
1 (SD NDVI)	*Aedes albopictus* abundance, ground index 1 , ground index 2, Aspect, roughness, TPI , SD canopy openness, distance to urban land , SD Temperature, Kurtosis Temperature, Kurtosis NDVI, Average EVI, SD EVI, Kurtosis EVI	210.06
2 (TPI)	*Aedes albopictus* abundance, ground index 1 , ground index 2, Aspect, roughness, SD canopy openness, distance to urban land , SD Temperature, Kurtosis Temperature, Kurtosis NDVI, Average EVI, SD EVI, Kurtosis EVI	208.37
3 (Ground Index 2)	*Aedes albopictus* abundance, ground index 1 , Aspect, roughness, SD canopy openness, distance to urban land , SD Temperature, Kurtosis Temperature, Kurtosis NDVI, Average EVI, SD EVI, Kurtosis EVI	205.03
4 (SD EVI)	*Aedes albopictus* abundance, ground index 1 , Aspect, roughness, SD canopy openness, distance to urban land , SD Temperature, Kurtosis Temperature, Kurtosis NDVI, Average EVI, Kurtosis EVI	203.86
5 (Kurtosis NDVI)	*Aedes albopictus* abundance, ground index 1 , Aspect, roughness, SD canopy openness, distance to urban land , SD Temperature, Kurtosis Temperature, Average EVI, Kurtosis EVI	**202.91***
6 (Ground Index 1)	*Aedes albopictus* abundance, Aspect, roughness, SD canopy openness, distance to urban land , SD Temperature, Kurtosis Temperature, Average EVI, Kurtosis EVI	**203.76***

*These two model are not statistically different (LRT= 2.852, df=1, P>0.09)

Table S4 Model selection for the best "full" zero inflated time series count model explaining the temporal abundance of *Aedes albopictus* 4th instar larvae and pupae in Mt. Konpira, Nagasaki – Japan. Fixed covariates indicate covariates that were considered in all models, while Changing covariates indicate pairs of covariates that where changes because they presented a high correlation (over 80%). Distribution indicates the count distribution. AIC indicates the Akaike Information Criterion, which is **bolded** for the best "Full" model, i.e., the one minimizing AIC. Lag for covariates is in biweeks. All models included water temperature (lag=0) as covariate for the zero inflation.

Fixed Covariates (Lag)	Changing Covariates (Lag)	Distribution	AIC
Auto-Regressive(1), Rainfall (2), SD of Rainfall (2), Relative Humidity (3), Kurtosis of Water Temperature (3), Kurtosis of Landsat based EVI (3), Kurtosis of MODIS based NDVI (1)	Water temperature (0), Landsat based NDVI (3)	Negative binomial	157.10
	Water temperature (0), Landsat based EVI (3)	Negative binomial	156.82
	Mean Air temperature (0), Landsat based NDVI (3)	Negative binomial	159.31
	Mean Air temperature (0), Landsat based EVI (3)	Negative binomial	159.61
	Minimum Air temperature (0), Landsat based NDVI (3)	Negative binomial	158.31
	Minimum Air temperature (0), Landsat based EVI (3)	Negative binomial	158.68
	Maximum Air temperature (0), Landsat based NDVI (3)	Negative binomial	158.92
	Maximum Air temperature (0), Landsat based EVI (3)	Negative binomial	159.37
	Water temperature (0), Landsat based NDVI (3)	Poisson	155.44
	Water temperature (0), Landsat based EVI (3)	Poisson	**155.25**
	Mean Air temperature (0), Landsat based NDVI (3)	Poisson	158.17
	Mean Air temperature (0), Landsat based EVI (3)	Poisson	158.36
	Minimum Air temperature (0), Landsat based NDVI (3)	Poisson	157.46
	Minimum Air temperature (0), Landsat based EVI (3)	Poisson	157.60
	Maximum Air temperature (0), Landsat based NDVI (3)	Poisson	157.41
	Maximum Air temperature (0), Landsat based EVI (3)	Poisson	157.70

Table S5 Model selection for the best "full" zero inflated time series count model explaining the temporal abundance of *Aedes flavopictus* 4th instar larvae and pupae in Mt. Konpira, Nagasaki – Japan. Distribution indicates the count distribution. AIC indicates the Akaike Information Criterion, which is **bolded** for the best "Full" model, i.e., the one minimizing AIC. Lag for covariates is in biweeks. All models included water temperature (lag=0) as covariate for the zero inflation.

Covariates (lag)	Distribution	AIC
Auto-regressive(1), relative humidity (0), Landsat based EVI (0), Kurtosis of Landsat based EVI (0)	Negative binomial	**298.16**
	Poisson	523.70

Table S6 Model Selection for the "best" zero inflated Poisson time series model explaining the temporal abundance of *Aedes albopictus* 4th instar larvae and pupae in Mt. Konpira, Nagasaki – Japan. AIC indicates the Akaike Information Criterion, which is **bolded** for the "best" model, i.e., the one minimizing AIC. Lag indicates the time lag in biweeks. All models included water temperature (lag=0) as covariate for the zero inflation.

Round (Removed covariate (lag))	Covariates (Lag)	AIC
0 (None removed)	Auto-Regressive(1), Rainfall (2), SD of Rainfall (2), Relative Humidity (3), Kurtosis of Water Temperature (3), Kurtosis of Landsat based EVI (3), Kurtosis of MODIS based NDVI (1), Water temperature (0), Landsat based EVI (3)	155.25
1 (Kurtosis of Landsat based EVI (3))	Auto-Regressive(1), Rainfall (2), SD of Rainfall (2), Relative Humidity (3), Kurtosis of Water Temperature (3), Kurtosis of MODIS based NDVI (1), Water temperature (0), Landsat based EVI (3)	153.30
2 (Relative Humidity (3))	Auto-Regressive(1), Rainfall (2), SD of Rainfall (2), Kurtosis of Water Temperature (3), Kurtosis of MODIS based NDVI (1), Water temperature (0), Landsat based EVI (3)	151.89
3(Landsat based EVI (3))	Auto-Regressive(1), Rainfall (2), SD of Rainfall (2), Kurtosis of Water Temperature (3), Kurtosis of MODIS based NDVI (1), Water temperature (0)	150.81
4(SD of Rainfall (2))	Auto-Regressive(1), Rainfall (2), Kurtosis of Water Temperature (3), Kurtosis of MODIS based NDVI (1), Water temperature (0)	149.97
5 (Rainfall (2))	Auto-Regressive(1), Kurtosis of Water Temperature (3), Kurtosis of MODIS based NDVI (1), Water temperature (0)	**149.25***
6 (Water temperature (0))	Auto-Regressive(1), Kurtosis of Water Temperature (3), Kurtosis of MODIS based NDVI (1)	**149.36***

*These two model are not statistically different (LRT= 1.030, df=1, P>0.3101)

Table S7 Model Selection for the "best" zero inflated negative binomial time series model explaining the temporal abundance of *Aedes flavopictus* 4th instar larvae and pupae in Mt. Konpira, Nagasaki – Japan. AIC indicates the Akaike Information Criterion, which is **bolded** for the "best" model, i.e., the one minimizing AIC. Lag indicates the time lag in biweeks. All models included water temperature (lag=0) as covariate for the zero inflation.

Round (Removed covariate (lag))	Covariates (Lag)	AIC
0 (None removed)	Auto-Regressive(1), relative humidity (0), Landsat based EVI (0), Kurtosis of Landsat based EVI (0)	298.16
1 (Kurtosis of Landsat based EVI (0))	Auto-Regressive(1), relative humidity (0), Landsat based EVI (0),	294.26
2 (relative humidity (0))	Auto-Regressive(1), Landsat based EVI (0),	**292.61**
